# Efficacy of Resection of Lateral Wall of Endolymphatic Sac for Treatment of Meniere's Disease

**DOI:** 10.3389/fneur.2022.827462

**Published:** 2022-03-11

**Authors:** Daogong Zhang, Yafeng Lv, Xiaofei Li, Yongdong Song, Ligang Kong, Zhaomin Fan, Haibo Wang

**Affiliations:** Department of Otolaryngology-Head and Neck Surgery, Shandong Provincial ENT Hospital, Cheeloo College of Medicine, Shandong University, Jinan, China

**Keywords:** Meniere's disease, vertigo, sensorineural hearing loss, resection of the lateral wall of the endolymphatic sac, surgery

## Abstract

**Background:**

To explore the long-term efficacy and safety of resection of the lateral wall of the endolymphatic sac for the treatment of intractable Meniere's disease (MD) as an alternative surgical procedure for treating this disorder.

**Methods:**

Data from 73 patients who were referred to our hospital and diagnosed with unilateral MD between January 2015 and June 2019 were retrospectively analyzed in this study. Seventy-three patients who had frequent vertigo even after receiving standardized conservative treatment for at least half a year underwent resection of the lateral wall of the endolymphatic sac. Vertigo control and auditory function were assessed. Pure tone audiometry, caloric test, and vestibular evoked myogenic potential were performed to evaluate audiological and vestibular functions. The post-operative follow-up duration was more than 2 years.

**Results:**

Among the 73 patients (male 34 cases, female 39 cases; age 20–69 years, average 51.4), vertigo was controlled effectively for 66 cases (90.4%) after 2 years of follow-up; 45 cases (61.6%) were completely controlled, and 21 cases (28.8%) were substantially controlled in this study. The patients of 16.4% had hearing loss with more than 10 dB change based on the four-tone average (0.5, 1, 2 and 3 kHz). No patient had a facial nerve weakness, cerebrospinal fluid leakage, or other complications.

**Conclusion:**

Resection of the lateral wall of the endolymphatic sac, which can effectively control vertiginous symptoms in intractable MD patients, represents an effective and safe therapy for this disease. Resection of the lateral wall of the endolymphatic sac is expected to be used as an alternative treatment for MD.

## Introduction

Meniere's disease (MD) is a common inner ear disorder characterized with intermittent episodes of vertigo, fluctuating sensorineural deafness, tinnitus, and/or aural pressure. Its prevalence ranges from 3.5% to 513 per 1,00,000 (1).There is currently no cure for this disease because its pathogenesis has not been established; ~80% patients can be free from vertigo after changes in lifestyle and medical treatment (1). Surgical procedures are considered when medical treatment fails to control vertigo. Endolymphatic sac surgery is widely used in patients with intractable MD; however, the rate of vertigo control is only 60–80%, and the benefit of this surgery is still debated (2–4). Vestibular neurectomy has a high rate of vertigo control, but it has several risks (5). Labyrinthectomy is only reserved as a last resort for those MD patients with total deafness (6).

In recent years, we have used resection of the lateral wall endolymphatic sac surgery to treat 73 cases of intractable MD and followed up for more than 2 years. The effectiveness and safety of this method were evaluated to provide a basis for its application to the treatment of intractable MD.

## Materials and Methods

### Patients

This study enrolled 73 patients (34 men, 39 women; age range 20–69 years, mean 51.4 years) diagnosed with ipsilateral MD according to the criterion by Barany society (7) and referred to vertigo clinic of our hospital between January 2015 and June 2019. The average course of these 73 patients was 72.8 months (24–480 months) (Table 1). All patients received standard medical treatment, consisting of betahistine 12 mg tid and hydrochlorothiazide 25 mg bid for over 6 months, but they continued to experience vertigo. All the patient had no migraine medical history and performed a battery of tests including auditory and vestibular examination and Gd-enhanced magnetic resonance imaging (MRI) to exclude the patients with vestibular migraine, cerebellopontine angle tumors or other intracranial space-occupying lesions. High resolution computerized tomography (CT) evaluation is performed before the surgery. Surgery is performed only when the anatomical conditions for endolymphatic sac surgery are available. All patients underwent resection of the lateral wall of the affected lymphatic sac. All patients were followed up for 2 years. The evaluation of treatment effects mainly focused on vertigo control and hearing change. The follow-up involved questionnaires, visits, and audiology and vestibular function examinations. Caloric test, vestibular evoked myogenic potential (VEMP) and pure tone audiometry were performed before and 2 years after the surgery.

**Table 1:** The demographic information and outcomes of patients with resection of the lateral wall of endolymphatic sac and endolymphatic sac decompression patients.

	**Resection of the lateral wall of endolymphatic sac**	**Endolymphatic sac decompression**	***P* value**
Sex			>0.05
Male	34	15	
Female	39	18	
Age	51.4	50.1	>0.05
Disease duration (months)	72.8	73.3	>0.05
Pure tone average (dB) before treatment	49.6	48.1	>0.05
Vertigo control rate (%)	90.4	72.7	<0.05
Hearing loss rate (%)	16.4	9.1	>0.05
Tinnitus improvement (%)	42.5	39.4	>0.05

To better evaluate the efficacy of endolymphatic sac resection, 33 patients who underwent endolymphatic sac decompression at the same time were allocated to the control group. They included 15 men and 18 women, with an average age of 50.1 years (22–70 years) and an average course of 73.3 months (18–444 months). Both the endolymphatic sac decompression and a lateral wall resection were offered to the MD patients with surgical indications. It completely depends on the patients' decision.

The study was approved by the Ethics Committee of Shandong Provincial ENT Hospital. All patients provided written informed consents.

### Surgical Procedures

Surgery was performed with a postauricular approach under general anesthesia.

The middle cranial fossa meninges, sigmoid sinus, sinus meningeal angle, and horizontal semicircular canal were exposed through a mastoidectomy. The endolymphatic sac was found between the sigmoid sinus and the posterior semicircular canal. The lateral wall of the endolymphatic sac was incised and a full blunt separation was performed between the inner wall and the outer wall. The isolated lateral wall of the endolymphatic sac was removed. After the bleeding was completely stopped, the incision was sutured and the surgery was completed.

### Evaluation of Vertigo

A definitive episode of vertigo lasting more than 20 min was regarded as Meniere's vertigo according to the American Academy of Otolaryngology-Head and Neck Surgery (AAO-HNS) criteria issued in 1995 (8). Patients were instructed to record acute spells of vertigo, coexisting symptoms (such as tinnitus, changes in hearing, aural fullness), and other characteristics, including time of onset and duration in a diary for the full 24 months of study. The average number of definitive spells in the last 6 months after therapy/average number of definitive spells in 6 months period before therapy × 100 = numeric value, where the numeric values are: 0 = complete control of definitive spells (class A); 1–40% = substantial control of definitive spells (class B); 41–80% = limited control of definitive spells (class C); 81–120% = insignificant control of definitive spells (class D); and >120% = worse control of definitive spells (class E). Effective vertigo control was defined as class A (complete control) and B (substantial control). After 2 years, patients with scale A and B were considered as having effective vertigo control according to the criteria of the AAO-HNS criteria issued in 1995.

### Evaluation of Hearing

Hearing was measured by a pure tone audiometer both using air- and bone-conducted pure-tone detection thresholds. The evaluation was based on the four-tone average (0.5, 1, 2, and 3 kHz) calculated according to the 1995 AAO-HNS criteria (8). The worst hearing level of the affected ear within the 6 months before surgery was compared with that between 18 and 24 months after surgery. Changes >10 dB were designated as “better” or “worse,” and changes <10 dB were designated as “no change” according to the 1995 AAO-HNS criteria.

### Evaluation of Tinnitus

According to the severity of tinnitus, tinnitus was divided into six grades. Grade 0, without tinnitus. Grade 1, occasionally tinnitus, but no pain; Grade 2, persistent tinnitus, worse when quiet; Grade 3, persistent tinnitus even in noisy environment; Grade 4, persistent tinnitus with attention and sleep disturbances; Grade 5: persistent severe tinnitus and unable to work; Grade 6, the patient is suicidal due to severe tinnitus. The improvement of tinnitus degree ≥1 has clinical significance.

### Caloric Test

The procedure of the bithermal caloric test had been reported on our previous study (9). Briefly speaking, each ear was irrigated with a constant flow of air at 24°C and 49°C for 40 s alternatively. The response was recorded over 3 min, and the interval of 7 min between the stimuli was allowed to prevent cumulative effects. A video-based system (Ulmer VNG, v. 1.4; SYNAPSYS, Marseille, France) was used to acquire and evaluate the eye response. The maximum slow-phase velocity of nystagmus after each irrigation was analyzed, and unilateral weakness (UW) was calculated. A UW value of <20% was considered normal.

### VEMP Test

The procedure had been reported on our previous study (9). Both cVEMP and oVEMP were tested from 2014 to 2017. Only cVEMP was tested before 2014.

Before 2014, A smart EP device (Intelligent Hearing Systems, USA) was used to record cVEMPs. The electromyographic activity of the sternocleidomastoid muscle (SCM) was recorded when patients lay supine and raise their head up to activate their neck flexors bilaterally. The saccular receptor was excited by air-conducted acoustic stimulation and the recording electrode was placed at the middle third of the SCM ipsilateral to the excited ear. The reference electrode was positioned at the upper edge of the sternum. The ground electrode was positioned at the muscle contralateral to the stimulated side. The amplifier gain was set to 100,000 and the bandpass and signals were filtered from 10 to 3,000 Hz. Short-tone bursts (100 dB n HL, 500 Hz) with a 1 ms rise-fall time and a 5 ms plateau time were delivered monaurally by TDH 49P earphones and the stimulation rate was 5 Hz. The duration of analysis was 60 ms and a total of 128 responses to stimuli were averaged. In order to check the test wave reproducibility, the measurements were repeated twice. A distinctly defined biphasic response was recorded on the SCM ipsilateral to the side of the cathode placement in all patients. We refer to this as p13/n23 response. The amplitudes of the p13-n23 and the peak latencies of p13 and n23 were analyzed. We obtained the averages of the amplitudes and latencies for the two runs. An amplitude ratio over 1.61 for the two ears was considered abnormal. The latencies of p13 exceed 17.3 ms and that of n23 exceed 24.6 ms were considered abnormal.

From 2014 to 2017, VEMPs were recorded by Neuro-Audio auditory evoked potential equipment (Neurosoft LTD, Ivanov, Russia). The test was done with the patients seated. Tone burst stimuli were delivered by an insert earphone (ER-3A). For the cVEMP examination, active recording electrodes were placed within the upper third region of the SCM on both sides. The reference electrodes were positioned on the upper sternum. The ground electrode was positioned on the nasion. The patient was asked to rotate the head toward the contralateral side of the stimulated ear to obtain tonic contraction of the SCM during recording. For the oVEMP examination, active recording electrodes were positioned on the infra-orbital ridge 1 cm below the center of each lower eyelid, and the reference electrodes were placed ~1 cm below them. A ground electrode was positioned on the nasion. The oVEMP was recorded with eyes open, maximally gazing upward. The stimulation rate was 5.1 Hz and the electrode impedance was maintained below 5 kΩ. We measured the VEMPs with a 500 Hz tone burst and the initial intensity was 110 dB nHL, decreased to the threshold in 5 dB steps. The cVEMP superimposition number was 60 and oVEMP superimposition number was 100 ≤ n ≤ 200. The duration of analysis was 0–50 ms and the bandpass filtering of cVEMP was 30–2,000 Hz. The bandpass filtering of oVEMP was 1–1,000 Hz. The latencies of p1 over 17.3 ms and the latencies of n1 over 24.6 ms of cVEMP were considered abnormal. An amplitude ratio over 30% was considered abnormal. The latencies of n1 over 12.6 ms and that of p1 over 17.8 ms of oVEMP were considered abnormal. An amplitude ratio more than 30% was considered abnormal.

### Statistical Analysis

The χ^2^ test and *t*-test were used to compare the demographic data of the patients who underwent lateral wall resection and sac decompression. *The χ*^2^ test was used to compare the vertigo control rates and hearing loss rates of patients who underwent lateral wall resection and those who underwent sac decompression. *p* < 0.05 was considered statistically significant.

## Results

In this work, the demographic information for the lateral wall resection and sac decompression groups are presented in Table 1. There were no significant differences in sex, age, disease course, and pre-operative hearing level between the two groups.

The total effective rate of vertigo control in the lateral wall resection group was 90.4% (66/73) at the 2-year follow-up, with a complete control rate of 61.6% (45/73) and a substantial control rate of 28.8% (21/73) (Table 1). The rate of hearing loss was 16.4% (12/73), hearing improvement was 8.2% (6/73), and hearing was unchanged in 75.3% (55/73). There was no significant difference between the patients with or without hearing loss (Supplementary Tables 1, 2). The tinnitus was improved in 31 cases (42.5%) and ineffective in 42 cases (57.5%).

Before surgery, 44 patients (60.3%) had abnormal caloric test results with poor responses on the affected side. Forty-six cases (63.0%) had an abnormal caloric test 2 years after treatment. Thirty-nine cases (53.4%) had abnormal cVEMP before the operation, with a decreased amplitude in the affected ear. Forty-one cases (56.2%) were abnormal 2 years post-operatively. Forty patients (54.8%) had abnormal oVEMP before surgery, with a decreased amplitude in the affected ear, and 38 cases (52.1%) were abnormal 2 years after the operation. There were no significant differences before and after the operation (Table 2; Supplementary Table 3). None of the patients had any complications, such as facial weakness or cerebrospinal fluid leakage.

**Table 2:** Abnormal rate of vestibular function tests pre- and post-operatively in resection of the lateral wall of endolymphatic sac group.

**Vestibular function tests**	**Pre-operative**	**Post-operative**	**χ^2^ Value**	***p* Value**
Caloric test	60.3%	63.0%	0.12	*p* > 0.05
cVEMP	53.4%	56.2%	0.11	*p* > 0.05
oVEMP	54.8%	52.1%	0.11	*p* > 0.05

The total control rate of vertigo in the endolymphatic sac decompression group was 72.7% (24/33), with a complete control rate of 45.5% (15/33) and a substantial control rate of 27.3% (9/33). The hearing loss rate was 9.1% (3/33). The tinnitus was improved in 13 cases (39.4%).

The vertigo control rate was significantly higher for the lateral wall resection group than for the endolymphatic sac decompression group (χ2 = 4.25, *p* < 0.05) (Table 1). There was no significant difference in the hearing loss rate and tinnitus improvement between the two groups (χ2 = 0.50, *p* > 0.05; χ2 = 0.016, *p* > 0.05) (Table 1).

## Discussion

There is currently no cure for MD; more than 85% of patients with these disorders benefit from changes in lifestyle or medical treatment. Surgery is considered when conservative treatment fails to control vertigo. In this study, we found that vertigo was controlled effectively in 90.4% of the 73 patients with MD who were treated with lateral wall resection of the endolymphatic sac at the 2-year follow-up. The rate of vertigo control with lateral wall resection of the endolymphatic sac was much higher than that of sac decompression in our study, suggesting that lateral wall resection of the endolymphatic sac is effective for the treatment of MD vertigo. However, the mechanism of action is not completely understood. We speculate the following. First, the endolymphatic sac plays a dual role in the formation of endolymphatic hydrops, which absorbs, as well as secretes, endolymph (10, 11). Nordström et al. found that 40% of human endolymphatic sac epithelial cells express Na + -K + -ATPase, indicating that they have considerable secretory ability (12). Immunohistochemical and ultrastructural studies showed that the endolymphatic sac of MD patients had excessive secretion of glycoprotein (13, 14), and the overexpression of aquaporin-2 in the endolymphatic sac epithelium was also involved in the formation of endolymphatic hydrops (15), suggesting that the endolymphatic sac secretion exceeded absorption, increasing the inner ear pressure. Li et al. used micro-computed tomography and high-resolution synchrotron phase contrast non-invasive imaging techniques to image and analyze the structures of the utricular duct and utricular-endolymphatic valve (or Bast's valve) of the human temporal bone specimens, suggesting that there is a two-way exchange of endolymphatic fluid involving the utricle, semicircular canal, and endolymphatic duct. Therefore, the authors speculated that vertigo associated with MD is caused by the sudden increase in endolymphatic pressure caused by the excessive secretion of the endolymphatic sac, which causes the endolymphatic fluid to flow back to the utricle and semicircular canal through Bast's valve (16). Resection of the lateral wall of the endolymphatic sac may reduce endolymphatic hydrops by reducing the secretion of endolymphatic fluid so that it can effectively control vertigo. Gibson et al. reported that 77 patients with MD were treated with partial resection of the lateral wall of the endolymphatic sac; 43 patients were followed up for 2 years, and the vertigo control rate was 83.7% (17). The effective rate in this study was slightly higher than that reported by Gibson. Daneshi et al. reported a new marsupialization technique in endolymphatic sac surgery. The outer layer of the sac was incised, turned around and placed under the anterior bony border, which is very similar with our ways in dealing with the sac wall. The vertigo control rate was evaluated by the vestibular score and deceased in 97.7% of the patients, which is consistent with our point on the vertigo control efficiency. However, there was no control group, only a pre- and post-operative control, and the inadequate evaluation of vertigo control, limiting the evidence effect in the study (18).

Second, the abnormal immune response of the endolymphatic sac may be the cause of MD. More and more studies have shown that the endolymphatic sac may be the “source” of inner ear immune response (19). Through high-throughput sequencing technology, we found that the expression of immune-related factors in the peripheral blood of MD patients was significantly higher than that in normal controls, suggesting that abnormal immune function may be involved in the pathogenesis of MD (20). Endolymphatic sac wall resection may eliminate or block the abnormal immune response of the endolymphatic sac to control vertigo attacks.

Our study also found that the effective rate of vertigo control after lateral wall resection of the endolymphatic sac significantly improved. The mechanism may be that endolymphatic sac wall resection may be more effective at eliminating or blocking the abnormal immune response of the endolymphatic sac than traditional endolymphatic sac decompression to control vertigo. After the resection of the lateral wall of the endolymphatic sac, the drainage of the endolymphatic fluid increased over a wider range, and the pressure of the membranous labyrinth was reduced, so the effect of controlling vertigo was better. The lateral wall resection of the endolymphatic sac is an enhancement of traditional endolymphatic sac surgery (such as endolymphatic sac decompression and drainage), which improves the control rate of vertigo.

Does excision of the lateral endolymphatic sac affect hearing? Our clinical data shows that hearing is preserved in more than 85% of patients, indicating that endolymphatic sac wall resection can preserve hearing. Prades et al., Darrouzet et al., and other scholars reported that hearing was still preserved with the retrolabyrinthine approach to resection of acoustic neuroma although the endolymphatic vessels were removed, indicating that the removal of the endolymphatic duct does not affect hearing (21, 22). The connection between the endolymphatic sac and the inner ear labyrinth is cut off after endolymphatic resection; therefore, it is speculated that hearing can be preserved after endolymphatic sac resection. Asmar et al. reported that patients who underwent endolymphatic blockage had the lateral wall of their endolymphatic sac removed at the same time, and there was no significant difference in post-operative hearing compared with patients with simple endolymphatic blockage (23). Gibson et al. reported that there was no significant decrease in hearing during the partial resection of the lateral wall of the endolymphatic sac, but ~56% of the patients had hearing loss during the 2-year follow-up. The authors believe that the cause of hearing loss may be related to the aggravation of membranous labyrinthine hydrops after surgery (17). However, Linthicum et al. reported that patients with endolymphatic sac resection did not develop membranous labyrinthine hydrops (24). In this study, approximately 16% of patients had hearing loss, which may be related to secondary labyrinth infection or aggravation of endolymphatic hydrops. In addition, hearing improved in four patients in this study during follow-up, which may be related to hearing fluctuations. Tinnitus was improved in approximately 40% of patients after resection of the lateral wall of the endolymphatic sac. The specific mechanism is not clear, which may be related to the reduction of membranous labyrinth hydrops and the relative stability or improvement of cochlear function. It may also be related to the effective control of vertigo, the reduction of psychological pressure, and the improvement of mental state and sleep.

In this study, the results of the caloric test and VEMP before and after resection of the lateral wall of the endolymphatic sac showed that this surgery had no significant effect on vestibular function. To date, no significant improvement in vestibular function has been observed with sac wall resection surgery. It is suitable for patients with bilateral vestibular dysfunction, especially those with bilateral MD. The current study is retrospective, and there are limitations in patient selection, control group and statistical methods.

## Conclusion

The etiology of MD has not been established. The current drug treatment is only to suppress the disease, and there is no complete radical cure. Our study shows that lateral wall resection of the endolymphatic sac is a safe and effective method for the treatment of intractable MD.

## Data Availability Statement

The raw data supporting the conclusions of this article will be made available by the authors, without undue reservation.

## Ethics Statement

The studies involving human participants were reviewed and approved by Ethics Committee of Shandong Provincial ENT Hospital. The patients/participants provided their written informed consent to participate in this study.

## Author Contributions

DZ and HW contributed to the conception of the work. DZ, ZF, and YL contributed to the experimental design. YL, XL, YS, and LK collected data and performed the analysis. All authors contributed to the interpretation of the data and were involved in writing the manuscript.

## Funding

This study was funded by National Natural Science Foundation of China (Nos. 82171150 and 81900940), Shandong Provincial Natural Science Foundation (No. ZR2020MH179) and Taishan Scholars Program of Shandong Province (No. ts20130913).

## Conflict of Interest

The authors declare that the research was conducted in the absence of any commercial or financial relationships that could be construed as a potential conflict of interest.

## Publisher's Note

All claims expressed in this article are solely those of the authors and do not necessarily represent those of their affiliated organizations, or those of the publisher, the editors and the reviewers. Any product that may be evaluated in this article, or claim that may be made by its manufacturer, is not guaranteed or endorsed by the publisher.
