# Supplementary material for: Efficacy of Resection of Lateral Wall of Endolymphatic Sac for Treatment of Meniere's Disease
Source: Front Neurol. 2022 Mar 11;13:827462. doi: 10.3389/fneur.2022.827462 (PMC8962735; doi:10.3389/fneur.2022.827462)
Supplement: Supplementary file 1 [file Table_1.docx]

Supplemental table 1. Vertigo control in the patients with or without hearing loss after endolymphatic sac decompression

|  |  | with hearing loss  (n=3) | without hearing loss(n=30) |
| --- | --- | --- | --- |
| Endolymphatic sac decompression | effective vertigo control | 2 | 22 |
|  | bad vertigo control | 1 | 8 |
| P | >0.05 |  |  |
