# Supplementary material for: Efficacy of Resection of Lateral Wall of Endolymphatic Sac for Treatment of Meniere's Disease
Source: Front Neurol. 2022 Mar 11;13:827462. doi: 10.3389/fneur.2022.827462 (PMC8962735; doi:10.3389/fneur.2022.827462)
Supplement: Supplementary file 3 [file Table_3.docx]

Supplemental table 3. Caloric test change before and after the resection of the lateral wall of endolymphatic sac surgery

|  |  | After surgery | |
| --- | --- | --- | --- |
|  |  | Abnormal | Normal |
| Before surgery | Abnormal | 44 | 0 |
|  | Normal | 2 | 26 |
